# Generation of flavor compounds by biotransformation of genetically modified hairy roots of *Hypericum perforatum* (L.) with basidiomycetes

**DOI:** 10.1002/fsn3.1573

**Published:** 2020-04-16

**Authors:** Hamide Ibrahimi, Sonja Gadzovska‐Simic, Oliver Tusevski, Arben Haziri

**Affiliations:** ^1^ Department of Chemistry Faculty of Natural Science and Mathematics University of Prishtina “Hasan Prishtina” Prishtina Kosovo; ^2^ Department of Plant Physiology Faculty of Natural Science and Mathematics Ss. Cyril and Methodius University Skopje North Macedonia

**Keywords:** basidiomycetes, flavor biogeneration, hairy roots, *Hypericum perforatum* (L.)

## Abstract

Altogether, 14 basidiomycetes (12BAD, 95PCH, 9WCOC, 5PSA, 96BCI, 331SHIBD, 4MSC, 74HFA, 220MPS, 115PFLA, 111 ICO C, 16LED, 6TSU, and 61LYP) were grown on solid and in liquid media using hairy roots of genetically modified *Hypericum perforatum* (L.) as the only source of carbon and nitrogen. After the first screening by GC‐MS/MS‐O, two fungi (115PFLA and 61LYP) which resulted in the most pleasant complex natural flavor by biotransformation were selected for further analysis. Twenty‐four new volatile compounds were produced, from which 21 were identified (ethyl hexanoate, ethyl octanoate, benzaldehyde, 2‐undecanone, (*E*,*E*)‐2,4‐decadienal, 1‐octen‐3‐one, (*E*)‐2‐nonenal, ethyl nonanoate, 2‐heptenal, 1‐methoxy‐4‐methylbenzene, 3‐octanone, 1‐decen‐3‐one, (*E*)‐2‐octenal, 1‐octen‐3‐ol, β‐linalool, ±trans‐nerolidol, anisole, methyl benzoate, 2‐pentylfuran, 1,3‐dichloro‐2‐methoxybenzene, and 1‐dodecanol). Thereof, 15 compounds were perceived at the ODP, from which 13 were identified. Compound identification was performed by comparison of Kovats indices (KI) and mass spectra to those of authentic reference compounds on a polar VF‐WAXms column using headspace solid‐phase microextraction–gas chromatography–mass spectrometry (HS‐SPME‐GC‐MS).

## 1 INTRODUCTION

The industrial interest for volatiles in general and especially for flavor compounds is increasing constantly (Guentert, 2007). Initially, flavor compounds have been extracted from plants and some animal sources. As they are typically present in low concentrations, extraction or other isolation methods are very laborious and expensive. Alternatively, they can be produced via chemical synthesis, but these compounds may not be labeled as “natural” (Vandamme & Soetaer, 2002). By developing of the industry, a lot of flavor compounds are producing via synthetic routs (Berger, 1995; Cheetham, 1993, 1997; Gatfield, 1997). Because of this, to consumers is developed chemophobia, expression used for compounds produced via chemical synthesis, even if they are nature‐identical (Berger, 1995; Cheetham, 1993, 1997; Gatfield, 1997). The use of chemical pathways for the synthesis of a flavor compound is often economically unsuitable, creates environmental contamination, or yield is not satisfactory due to the formation of racemic mixtures. According to the US and European regulations, a natural flavor may be obtained via microbial or enzymatic processes, provided that the precursor (raw material) is also natural and obtained via physical or biotechnological processes.

As mentioned above, flavor compounds can be isolated from plant material by using a different organic solvent and methods, but unfortunately their concentration sometimes is lower than 0.01 g/L (Gounaris, 2010). As the industry needs are far bigger than that, synthetic manufacture of volatiles and flavor compounds is growing day by day, like vanilla, for example, 9/10 is coming by synthetic product (Dignum, Kerler, & Verpoorte, 2007). Production of flavor compounds via fermentation or biocatalysis means production via bio routes, during which the product is natural. Flavor compounds resulting from fermentation or biocatalysis are organic products. Synthetic counterparts, compared to natural, have lower price but costumers are hesitating to use them (Janssens, Pooter, Schamp, & Vandamme, 1992). Biotechnological processes usually use conditions that are less harmful to the environment and the yields of flavor compounds are much higher than those found in, for example, fruit or vegetables (Vandamme & Soetaer, 2002). Considering all of this factors, biotechnological production of volatiles and flavor compounds by involving fungi, yeasts, and bacterial cultures results in “natural flavor compounds” (Gounaris, 2010). Using traditional starter cultures in biotechnological processes for fermentation of by‐products is often not sufficient, for example, the spectrum of aroma compounds formed by yeasts of the genus *Saccharomyces* is limited (Carrau et al., 2008). Since the beginning of the 1950s, attempts have been made to utilize the great biochemical potential of higher fungi to produce various natural flavor compounds (Sugihara & Humfeld, 1954). Basidiomycetes are well known for their nutritional properties and their often pleasant taste for thousands of years (Lorenzen & Anke, 1998), and because of their unique system of extracellular enzymes, also known as secretome, they are able to produce different pharmacologically relevant secondary metabolites and flavor compounds de novo or by biotransformation (Bouws, Wattenberg, & Zorn, 2008; Fraatz & Zorn, 2011). As complex organisms, fungi were studied for so long by chemists, biochemists, genetics, ecologist, biologist, and so on (Tkacz & Lange, 2004). Using fungi for production of traditional products have a long tradition (Papagianni, 2004). Production of flavor compounds from microorganism was started at early of 1980s, and their importance as a big source for this production was academically proved in between 30 and 40 years before (Gatfield, 1999). As a mayor class of fungi, basidiomycetes are well known for their nutritional properties and their often pleasant taste for thousands of years (Lorenzen & Anke, 1998). Generation of flavor compounds from fungi and their perspective in flavor production by biotechnological processes have been well described by several authors (Agrawal, 2004; Berger & Zorn, 2004; Vandamme, 2003). *Pleurotus sapidus,* for example, has been shown to efficiently transform the sesquiterpene hydrocarbon valencene to nootkatone, which is an important contributor to the flavor of grapefruit (Bouws et al., 2008).

*Hypericum perforatu*m (L.) known also as Saint John's wort has been used for decades because of its pharmaceutical attributes (Nahrstedt & Butterweck, 2010). In order to avoid the influence of external factors (temperature, pollution, climate changes, etc.) on the quality and quantity of the active secondary metabolites of plant material, scientists are using the genetic transformation (Tusevski et al., 2013). The first successful transformation of *H. perforatum* (L.) was done using *Agrobacterium rhizogenic* ATCC 15,834 (Di Guardo et al., 2003). The transformation protocol and establishment of hairy roots material used for this study was proved using PCR analysis (Tusevski et al., 2013). The application of this transformation verifies the production of some new phenolic compounds with important role as secondary metabolites with pharmaceutical activity of the hairy roots (Tusevski et al., 2013). By knowing the nature of the secondary metabolites, which are produced by the hairy roots of the genetically modified (GMO) *H. perforatum* (L.), we decided to use these hairy roots as unique and special material for bio‐production of new flavor compounds with basidiomycetes. We decided to use roots part of genetically modified *H. perforatum* (L.) because after sniffing they had only a few flavor compounds compared with aerial part (shoots) which seemed to have many more, what means that we have used a raw material with few flavor compounds, to produce variety classes of flavor compounds using basidiomycetes.

## 2 EXPERIMENTAL

### 2.1 Substrates

Hairy roots of GMO *H. perforatum* (L.) were provided from the Department of Plant Physiology at Ss. Cyril and Methodius University (North Macedonia). Roots of GMO *H. perforatum* (L.) were stored in −20°C until used. Prior to the biotransformation experiments, the roots were homogenized with liquid nitrogen.

### 2.2 Microorganisms

Fourteen different basidiomycetes were obtained from the Centraalbureau voor Schimmelcultures (CBS, Utrecht, Netherlands), the German Collection of Microorganisms and Cell Cultures (DSMZ, Braunschweig, Germany) and the Department of Molecular Wood Biotechnology and Technical Mycology (WBTM, Göttingen, Germany).

### 2.3 Chemicals

Authentic flavor standards were purchased from Sigma‐Aldrich, Fisher Scientific, Alfa Aesar, TCI Deutschland, Th. Geyer, and VWR. From the 24 volatile compounds formed during this study, 19 were identified by comparison to authentic reference standards on a VF‐WAXms column by GC/MS‐MS/O (ethyl hexanoate, ethyl octanoate, benzaldehyde, 2‐undecanone, (*E*,*E*)‐2,4‐decadienal, 1‐octen‐3‐one, (*E*)‐2‐nonenal, ethyl nonanoate, 3‐octanone, 1‐decen‐3‐one, (*E*)‐2‐octenal, 1‐octen‐3‐ol, β‐linalool, ±trans‐nerolidol, anisole, methyl benzoate, 2‐pentylfuran, 1,3‐dichloro‐2‐methoxybenzene, and 1‐dodecanol). 2‐Heptenal and 1‐methoxy‐4‐methylbenzene were tentatively identified by comparison of their Kovats indices and mass spectra with those of the NIST 2011 database, while three other compounds were not identified. All chemicals were of analytical grade.

### 2.4 Submerged cultures

*Substrate preparation.* Hairy roots of GMO *H. perforatum* (L*.*) obtained from Skopje University (Tusevski et al., 2013) were used as a substrate for the submerged cultures. Hairy root material was homogenized under liquid nitrogen and stored at −20°C.

*Precultures and fermentation of substrate. Bjerkardera adusta* (12BAD), *Phanerochaete chrysosporium* (95PCH), *Wolfiporia cocos* (9WCOC), *Pleurotus sapidus* (5PSA), *Botrycis cineraea* (96BCI), *Stereum hirsutum* (331SHI‐BD), *Mycetinis scorodonius* (4MSC), *Hypholoma fasciculare* (74HFA), *Mycena pseudocorticola* (220MPS), *Pleurotus flabellatus* (115PFLA), *Irpex consors* (111ICO C), *Lentilula edodes* (16LED), *Trametes suaveolens* (6TSU), and *Lycoperdon pyriforme* (61LYP) were inoculated into Standard Nutrition Solution (SNS) which contained: 30 g/L glucose monohydrate, 4.5 g/L asparagine monohydrate x H_2_O, 1.5 g/L mono potassium phosphate (KH_2_PO_4_), 0.5 g/L magnesium sulfate nH_2_O (MgSO_4_ × H_2_O), 3 g/L yeast extract, 1 ml/L trace element solution (CuSO_4_ × 5 H_2_O 5 mg/L; FeCl_3_ × 6 H_2_O 80 mg/L; MnSO_4_ × H_2_O 30 mg/L; ZnSO_4_ × 7 H_2_O 90 mg/L; EDTA 0.4 g/L) (Zhang, Fraatz, Horlamus, Quitmann, & Zorn, 2014). The pH of the SNS was adjusted to 6 by adding sodium hydroxide solution (1 M) or hydrogen chloride (1 M) prior to sterilization. The medium was autoclaved for 20 min at 121°C. Erlenmeyer flasks with solution (100 ml /250 ml, medium volume/flask volume) were incubated on a rotary shaker (24°C, 150 rpm/min, 25 mm shaking diameter in the dark) until sufficient growth was observed. The mycelium of 10 ml preculture was precipitated using centrifugation with 4,000 rpm (2,150× *g*) for 10 min at 24°C. The mycelium was washed three times with sterile water, and the pellets were re‐suspended in 10 ml of sterile water. The suspension was added into an Erlenmeyer flask (250 ml) containing 100 ml of *hairy roots* medium (10 g/L). For comparison, three blank samples were prepared, I) only plant with water, II) fungus with minimal medium, and III) fungus with sterile water. The minimal medium (MM) solution contained 6.24 g/L mono sodium‐L‐aspartate × 1 H_2_O, 3 g/L glucose, 2.4 g/L ammonium nitrate (NH_4_NO_3_), 1.5 g/L potassium hydrogen phosphate (KH_2_PO_4_), and 1 ml/L trace element solution (CuSO_4_ × 5 H_2_O 5 mg/L; FeCl_3_ × 6 H_2_O 80 mg/L; MnSO_4_ × H_2_O 30 mg/L; ZnSO_4_ × 7 H_2_O 90 mg/L; EDTA 0.4 g/L). The fermentation was carried for 8 days during which samples were taken daily and analyzed by sensory evaluation.

### 2.5 Sensory evaluation of cultures

Liquid samples containing supernatant, roots of GMO *H. perforatum* (L*.*), and mycelium were taken daily and transferred into odorless medicinal cups (5 ml sample/20 ml volume of cup). The odor impression was recorded by a panel of five trained panelists. The odor intensity was rated on a scale of one to four (1: low intensity of flavor ‐ 4: intense, strong flavor).

### 2.6 Surface screening

Roots of GMO *H. perforatum* (L*.*) were autoclaved at 121°C for 20 min separately in a small Erlenmeyer flask covered with aluminum foil. Solution of MEA 2% (2 g agar‐agar/100 ml H_2_O) was autoclaved and mixed with the roots under sterile conditions. The suspension was poured into small Petri dishes. The plates were inoculated with the fungi mentioned above, and the growth was evaluated visually daily. When about 70% of the surface of the Petri dishes was covered by fungal mycelium, the surface cultures were sniffed by the trained panel; the screening process has been repeated three times.

### 2.7 Headspace solid‐phase microextraction (HS‐SPME)

For HS‐SPME, the DVB/CAR/PDMS and PDMS/DVB fibers were evaluated. After comparison of the respective chromatograms, PDMS/DVB fibers were chosen for further analyses because of their higher sensitivity (polydimethylsiloxane/divinylbenzene; film thickness 75 μm, fiber length 1 cm; Supelco, Steinheim, Germany) in combination with a GERSTEL MPS 2XL multi‐purpose sampler (GERSTEL, Mülheim and der Ruhr, Germany).

Ten mL samples from submerged cultures were transferred into a sterilized headspace vial (20 ml) and capped. The sample was agitated for 10 min (250 rpm) at 40°C followed by headspace extraction for 30 min at the same temperature. The analytes adsorbed on the fiber were directly desorbed in the split/splitless inlet (250°C; SPME liner, 0.75 mm i.d.; Supelco) of the GC/MS‐MS/O system for 1 min. The fiber was moved to the SPME fiber conditioning station and heated at 250°C for 20 min.

### 2.8 Gas Chromatography‐olfactometry (GC‐MS/MS‐O)

Gas chromatography apparatus used for all measurements was from Agilent Technologies, (Waldbronn, Germany) 7890A equipped with an Agilent 7000B triple quadrupole mass spectrometry (MS/MS) detector (Agilent Technologies). A polar Agilent VF‐WAXms column (30 m × 0.25 mm i.d.  × 0.25 μm film thickness, Agilent Technologies) was used for analysis. Helium was used as carrier gas at a constant flow rate of 1.56 ml/min. At the end of the column, the carrier gas was split 1:1 into the triple quadrupole mass spectrometer and into an olfactory detector port (ODP 3, GERSTEL, Mülheim an der Ruhr, Germany). Temperature of the program 40°C (3 min)/5°C/min to 240°C (5 min); injection temperature 250°C; septum purge flow rate, 3 ml/min; MS modes, scan mode in Q1; scan range *m/z* 33–330; electron ionization energy, 70 eV; source temperature, 230°C; quadrupoles temperature, 150°C; MS/MS transfer line temperature 250°C; He quench gas, 2.25 ml/min; N_2_ collision gas, 1.5 ml/min; ODP 3 transfer line temperature, 250°C; ODP 3 mixing chamber, 150°C; ODP 3 make up gas, N_2_. GC‐MS/MS analyses were performed using an Agilent 7,890 gas chromatograph from Agilent Technology, equipped with a model 5975C mass spectrometry detector, also from Agilent Technology (carrier gas, helium; constant flow rate, 1.2 ml/min; inlet temperature, 250°C; split ratio, 10:1; septum purge flow rate, 3 ml/min; 30 m x 0.25 mm i.d., 0.25 μm Agilent J&W DB‐5MS column; scan range, *m/z 33–330*; electron ionization energy, 70 eV; source temperature, 230°C; quadrupole temperature, 150°C; transfer line temperature, 250°C). The condition used in this research was in accordance with a scientific work conducted by Zhang et al., (2014).

### 2.9 Identification of flavor compounds

Identification of flavor compounds was performed by comparison of Kovats indices (KI), odor impressions, and mass spectra to those of authentic reference compounds and with the NIST 2011 MS library database.

## 3 RESULTS AND DISCUSSION

**FIGURE 1:**
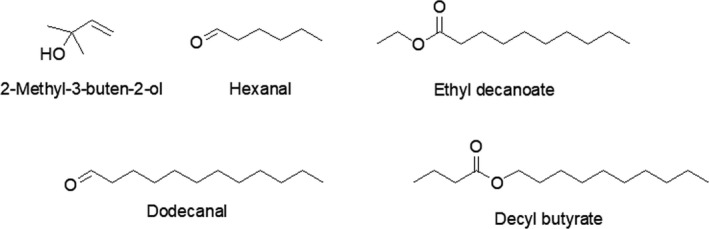
Chemical structures of tentatively identified odor active compounds of hairy roots of GMO *H. perforatum* (L*.*)

The volatiles emitted from hairy roots from GMO *H. perforatum* (L.) were analyzed by GC‐MS/MS and GC‐MS/MS‐O at 40°C (3 min)/ 5°C/min to 240°C (5min), and five compounds (2‐methyl‐3‐buten‐2‐ol, hexanal, ethyl decanoate, dodecanal, decyl butyrate) were tentatively identified. All of these compounds were detected with low peak intensities only (Table 1, Figure 1).

**TABLE 1:** Tentatively identified flavor compounds of hairy roots of GMO *H. perforatum* (L*.*). (n.i: not identified)

Identified flavor compound	Odor description	Odor intensity	KI (VF‐WAXms)	KI‐NIST 2011	Identification
2‐Methyl‐3‐buten‐2‐ol	Unknown	1	1,038	1,036	MS, KI
Hexanal	Fruity	1	1,077	1,078	MS, KI, odor
n.i	Unknown	1	1,297		
n.i	Fresh	2	1,422		
n.i	Unknown	2	1,496		
Ethyl decanoate	Sweet	2	1634	1643	MS, KI, odor
Dodecanal	Fresh	3	1705	1709	MS, KI, odor
Decyl butyrate	Fresh	2	1817	1807	MS, KI, odor
n.i	Fresh	2	1855		
n.i	Unknown	1	2,213		

### 3.1 Surface screening

In the surface screening, two of the 14 strains evaluated in the screening exhibited interesting flavor profiles (Table 2).

**TABLE 2:** Selected basidiomycetes from surface screening and their impressions

Basidiomycete	Sensory impression
115PFLA	Cucumber, green vegetable, boiled vegetable
61LYP	Fresh, seed

*Submerged cultures.* All 14 basidiomycetes (12BAD, 95PCH, 9WCOC, 5PSA, 96BCI, 331SHIBD, 4MSC, 74HFA, 220MPS, 115PFLA, 111 ICO C, 16LED, 6TSU, and 61LYP) were screened in submerged cultures. Sensory analysis was performed daily by heating an aliquot of the culture (5 ml) at 40°C for a few minutes in a sterile cup. In parallel, three blank samples were evaluated. Changes of the sensory profile were described as pleasant, unpleasant, and odorless. In most cases, the flavor impressions were similar to those of the respective surface cultures. For example, 115PFLA and 61LYP showed pleasant flavors. However, differences were observed with 12 BAD. This strain exhibited a fresh and fruit aroma in the surface screening, which was not observed during olfactory evaluation of the liquid cultures. Similarly, 9WCOC was perceived as fresh and orange‐like in surface cultures, while no specific odor was detected for the submerged cultures.

*Screening by measuring on GC‐MS/MS‐O.* From the 14 basidiomycetes cultivated in liquid media, 2 strains (115PFLA and 61 LYP) which resulted in the formation of the most pleasant flavor compounds were selected for detailed analysis of the flavor profiles by GC‐MS/MS‐O at 40°C (3 min)/ 5°C/min to 240°C (5min). Evaluation by GC‐MS/MS‐O was done daily, and new volatile compounds were formed depending on the respective culture period. All results were reproduced twice.

### 3.2 Flavor analysis

Microorganisms are able to produce all classes of volatile compounds, but aldehydes, alcohols, and organic acid esters are often dominating. In general, biotransformation products were observed between 24 and 190 hr (Gounaris, 2010).

**FIGURE 2:**
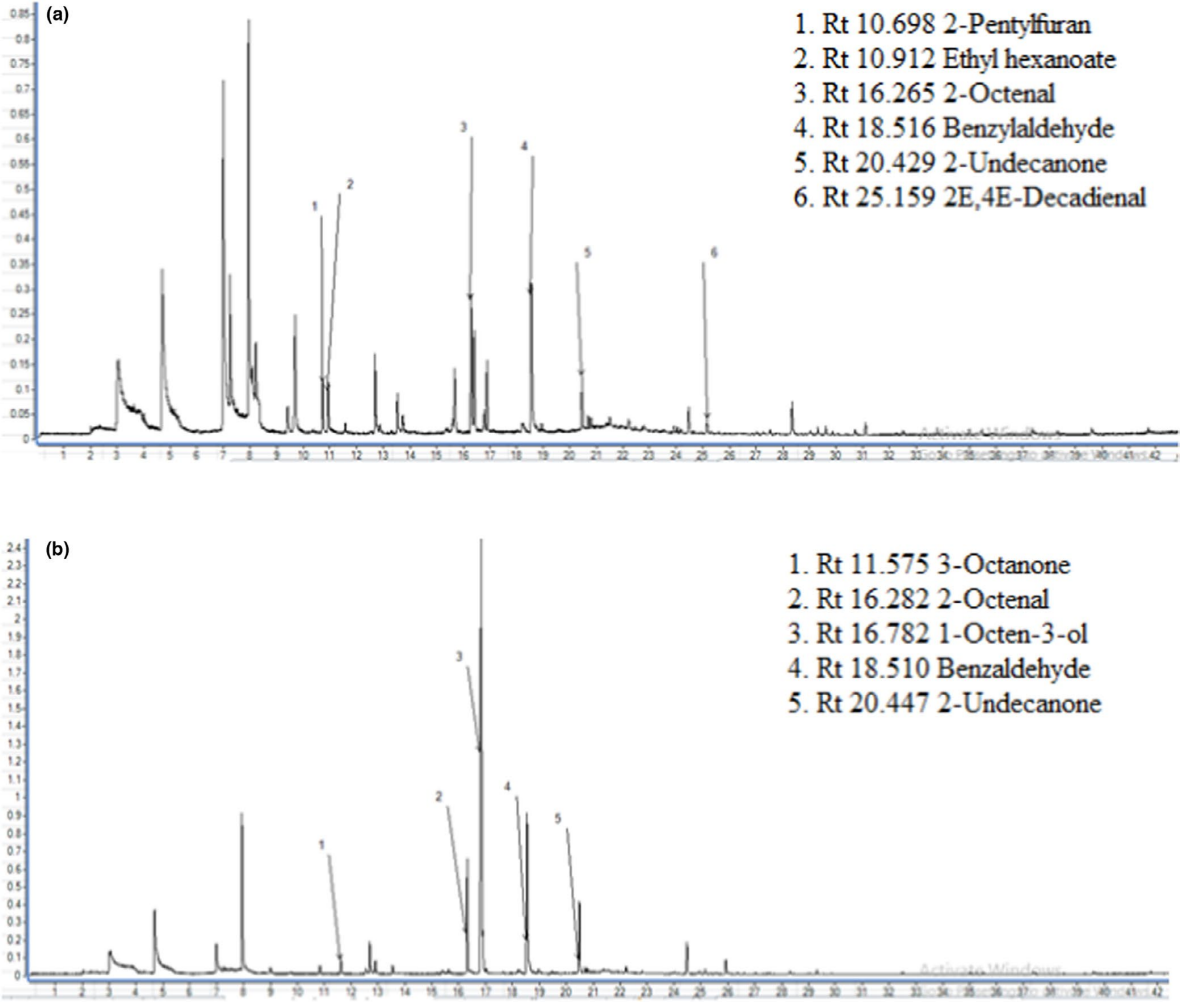
Comparison of the chromatograms of the biotransformation of hairy roots of *H. perforatum* (L.) by 115 PFLA obtained on different culture days: a) day 1; b) day 8

*Pleurotus flabellatus* (115PFLA) produced mostly aldehydes (benzaldehyde, (*E*,*E*)‐2,4‐decadienal, 2‐nonenal, 2‐heptenal), esters (ethyl hexanoate, ethyl octanoate, ethyl nonanoate), ketones (2‐undecanone, 1‐octen‐3‐one), an anisole derivate (1‐methoxy‐4‐methylbenzene), and two nonidentified compounds. From the twelve volatiles produced by 115PFLA, eight were detected at the ODP as flavor compounds (ethyl octanoate, (*E*,*E*)‐2,4‐decadienal, 1‐octen‐3‐one, 2‐nonenal, ethyl nonanoate, 2‐heptenal, 1‐methoxy‐4‐methylbenzene, and one nonidentified compound). All compounds produced by 115PFLA are listed in Table 3. The results presented in Table 3 and Figure 2 indicate dynamic changes of the flavor profiles over the culture period.

**TABLE 3:** Identification of compounds formed by *P. flabellatus* (115PFLA)

Compounds^a^	Fermentation time^b^/h	Odor description^c^	Odor Intensity^d^	KI (VF‐WAXms)	Identification
Ethyl hexanoate	24	Pineapple	1	1,223	MS, KI, odor
Ethyl octanoate	24	Banana	2	1,427	MS, KI, odor
Benzaldehyde	24, 44, 68, 93, 117, 141, 165, 189	No odor	1	1,511	MS, KI
2‐Undecanone	24, 44, 68, 93, 117, 141, 165, 189	No odor	1	1,591	MS, KI
(*E*,*E*)‐2,4‐Decadienal	24, 68	Fresh	2	1,800	MS, KI, odor
n.i.	44, 68, 117, 141, 189	No odor	1	1,179	
1‐Octen−3‐one	117,165	Green	3	1,294	MS, KI, odor
*n*.i	68	Fresh	4	1,637	
(*E*)−2‐Nonenal	189	Cucumber	3	1,525	MS, KI, odor
Ethyl nonanoate	24	Fresh	4	1,528	MS, KI, odor
2‐Heptenal	44	Green‐fatty	2	1,317	MS, KI, odor
1‐Methoxy‐4‐methylbenzene	44	Fruity	2	1,397	MS, KI, odor

Abbreviations: 2: low intensity; 3: middle intensity; 4: high intensity.

^a^ compounds considered as formed de novo or by biotransformation.

^b^ Fermentation time indicates that some compounds may be found in the biotransformed product in different hours.

^c^ Odor description defined during olfactory detection.

^d^ Odor intensity from 1–4 (1: no odor; n.i: Not identified).

*Lycoperdon pyriforme* (61LYP) is able to produce a variety of compounds comprising ketones (3‐octanone, 1‐decen‐3‐one, 2‐undecanone), esters (ethyl octanoate, ethyl hexanoate, 1‐methoxy‐4‐methylbenzene), alcohols (1‐octen‐3‐ol, 1‐dodecanol), a terpene alcohol (β‐linalool), an aldehyde ((*E*)‐2‐octenal), a chlorinated compound (1,3‐dichloro‐2‐methoxybenzene), a sesquiterpene (±trans‐nerolidol), a furan derivate (2‐pentylfuran), an aromatic ether (anisole), and one not identified (n.i.) compound. From the 15 new volatile compounds produced during biotransformation, seven were perceived at the ODP, from which six were identified (1‐decen‐3‐one, (*E*)‐2‐octenal, 1‐octen‐3‐ol, β‐linalool, ±trans‐nerolidol, and 1,3‐dichloro‐2‐methoxybenzene) and one n.i. compound. All volatile compounds produced by 61LYP are summarized in Table 4. Compounds as ethyl octanoate, 2‐undecanone, and ethyl hexanoate were also biosynthesized by 115PFLA.

**TABLE 4:** Biotransformation compounds formed by *L. pyriforme* (61LYP)

Compounds^a^	Fermentation time^b^/h	Odor description^c^	Odor intensity^d^	KI (VF‐WAXms)	Identification
3‐Octanone	24, 48, 144, 168, 192	No odor	1	1,248	MS, KI
1‐Decen‐3‐one	24, 48, 72, 96, 168, 192	Green	3	1,294	MS, KI, odor
(*E*)‐2‐Octenal	24, 48, 96, 168	Fresh‐herbal	3	1,423	MS, KI, odor
Ethyl octanoate	24	No odor	1	1,428	MS, KI
1‐Octen‐3‐ol	24, 48	Green	2	1,443	MS, KI, odor
β‐Linalool	24, 48, 72, 96, 144, 168, 192	Herbal, green	4	1,540	MS, KI, odor
2‐Undecanone	24, 48, 96, 144, 192	No odor	1	1,595	MS, KI
(±)trans‐Nerolidol	24, 48	Seeds	2	2,029	MS, KI, odor
Ethyl hexanoate	48, 72	No odor	1	1,227	MS, KI
Anisole	72	No odor	1	1,334	MS, KI
Methyl benzoate	72	No odor	1	1611	MS, KI
2‐Pentylfuran	96, 144, 168, 192	No odor	1	1,215	MS, KI
1,3‐Dichloro‐2‐methoxybenzene	96, 192	Medicinal	2	1,691	MS, KI, odor
1‐Dodecanol	144, 192	No odor	1	1,957	MS, KI
n.i	144, 192	Seeds	2	2,021	

Abbreviations: 2: low intensity; 3: middle intensity; 4: high intensity.

^a^ compounds considered as formed de novo or by biotransformation.

^b^ Fermentation time indicates that some compounds may be found in the biotransformed product at different time points.

^c^ Odor description defined during olfactory.

^d^ Odor intensity from 1–4 (1: no odor; n.i: Not identified).

Some C‐8 compounds are well known as fungal volatiles, originating from the enzymatic oxidation of linoleic acid. 1‐Octen‐3‐one from 115PFLA, 2‐octenal, and 1‐octen‐3‐ol from 61 LYP were not found in the blank samples which indicates that they are biosynthesized as flavor compounds (Zhang et al., 2014).

**FIGURE 3:**
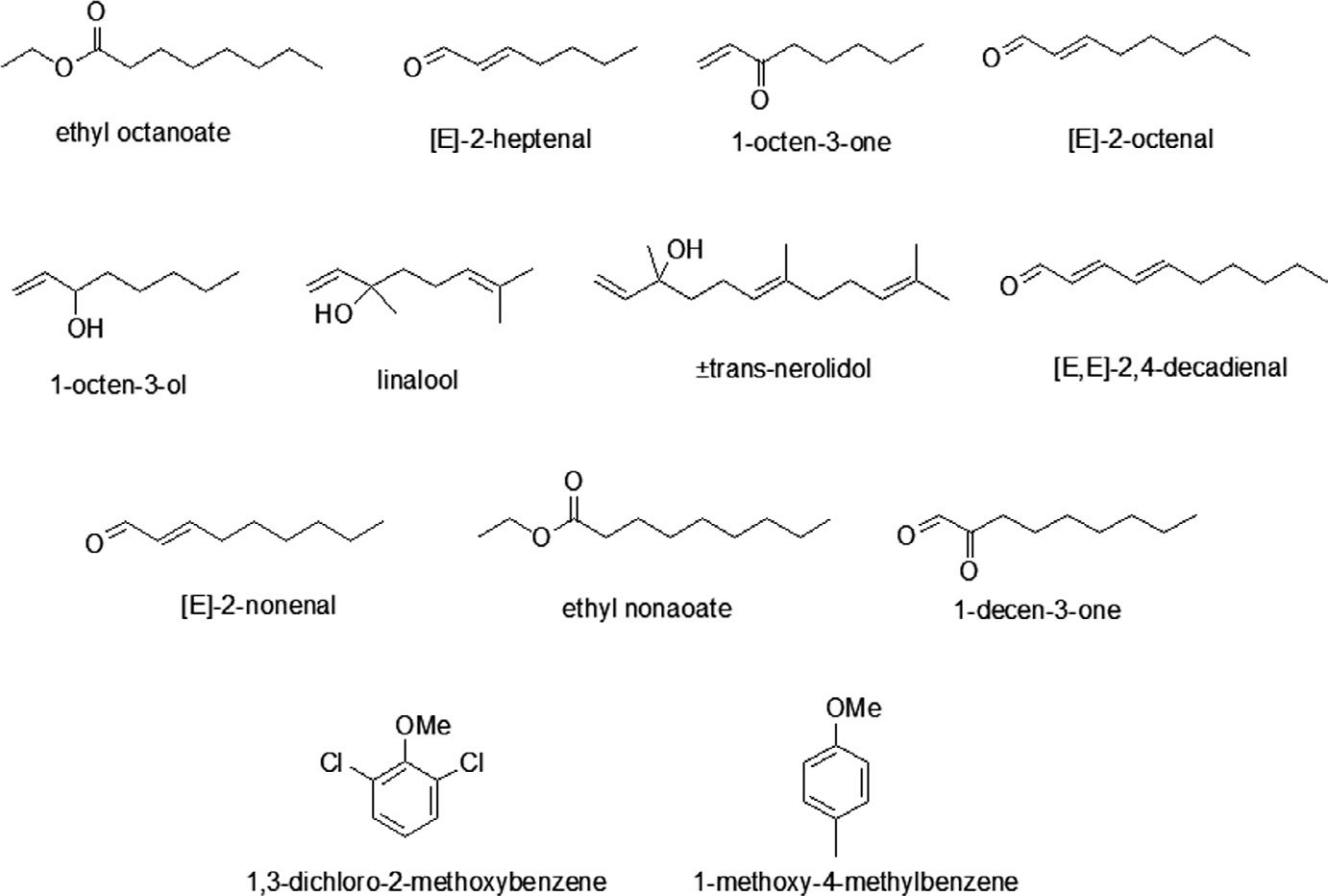
Chemical structures of the identified odor active compounds

The two selected basidiomycetes produced different classes of compounds, with a total of 24 new volatile compounds, from which 17 were identified using injection of analytical standards, calculation of Kovats indices and comparing their values with the NIST 2011 database together with their mass spectra (Tables 3 and 4). Two compounds were identified only by injection of analytical standards, calculation Kovats Index values and comparing values because there were no data on NIST 2011 database, 2 others only by comparing values with the NIST 2011 database, and three compounds were not identified. From the total 24 new volatile compounds, 15 were flavor compounds (ethyl octanoate, (*E*,*E*)‐2,4‐decadienal, 1‐octen‐3‐one, (*E*)‐2‐nonenal; ethyl nonanoate, 2‐heptenal, 1‐methoxy‐4‐methylbenzene, 1‐decen‐3‐one, (*E*)‐2‐octenal, 1‐octen‐3‐ol, β‐linalool, (±)trans‐nerolidol, 1,3‐dichloro‐2‐methoxybenzene, and 2 nonidentified flavor compounds). The structures of these compounds are shown in Figure 3.

## 4 CONCLUSIONS

By treating roots of GMO *H. perforatum* (L.) with basidiomycetes, we aimed to produce new volatile compounds by focusing in flavor ones. As the result of this study, 24 new volatile compounds were formed, from which 15 seems to be flavor compounds. Altogether, 21 compounds were identified, from which 17 by injection of analytical standard and comparing Kovats Index values together with mass spectra with NIST 2011 database, (ethyl hexanoate, ethyl octanoate, benzaldehyde, 2‐undecanone, (*E*,*E*)‐2,4‐decadienal, 1‐octen‐3‐one, (*E*)‐2‐nonenal, ethyl nonanoate, 3‐octanone, (*E*)‐2‐octenal, 1‐octen‐3‐ol, β‐linalool, ±trans‐nerolidol, anisole, methyl benzoate, 2‐penthylfuran, 1‐dodecanol), two other compounds were identified only by injection of analytical standards (1,3‐dichloro‐2‐methoxybenzene and 1‐decen‐3‐on) because there are no data in NIST 2011 database and two others (2‐heptenal and 1‐methoxy‐4‐methylbenzene) only by comparing Kovats Index values with NIST 2011 database, because this compounds were not found as analytical standard and three other compounds were not identified. In all cases, cultures were extracted using headspace solid‐phase microextraction coupled with double gas chromatography–mass spectrometry–olfactory (HS‐SPME‐GC/MS‐MS‐O).

## CONFLICT OF INTEREST

The authors declare that there are no conflicts of interest to disclose.

## ETHICAL APPROVAL

There was no human or animal testing in this study.
