# Supplementary figures and images for: Generation of flavor compounds by biotransformation of genetically modified hairy roots of Hypericum perforatum (L.) with basidiomycetes
Source: Food Sci Nutr. 2020 Apr 16;8(6):2809–16. doi: 10.1002/fsn3.1573 (PMC7300056; doi:10.1002/fsn3.1573)

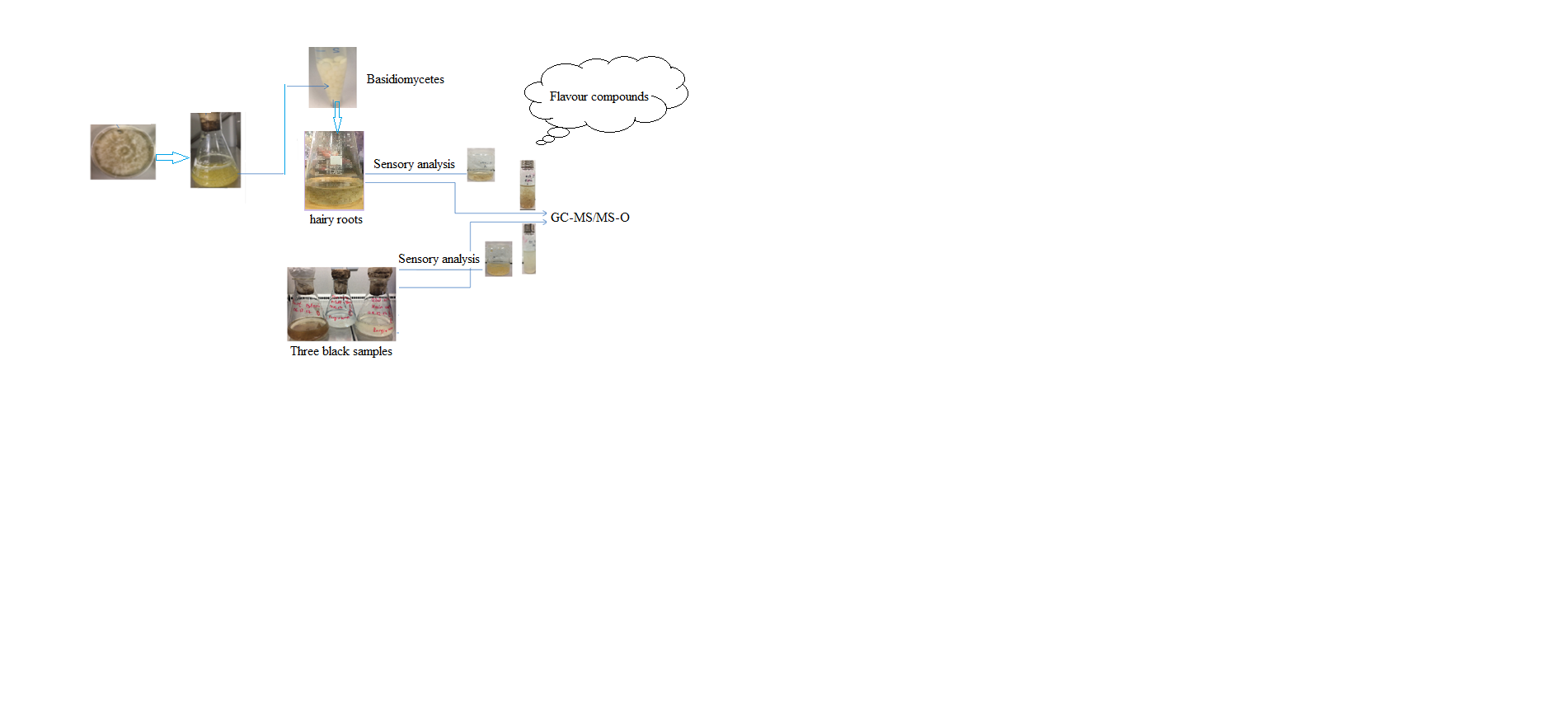

Supplement: Supplementary file 1 [file FSN3-8-2809-s001.tif]
